# Reassembling of albumin-bound paclitaxel mitigates myelosuppression and improves its antitumoral efficacy via neutrophil-mediated targeting drug delivery

**DOI:** 10.1080/10717544.2022.2046892

**Published:** 2022-03-04

**Authors:** Yuxin Chen, Lulu Han, Xiaoyan Qiu, Meng Wang, Zheng Chen, Ying Cai, Yong Xin, Yanfang Lv, Ankang Hu, Dafei Chai, Liantao Li, Huizhong Li, Junnian Zheng, Gang Wang

**Affiliations:** a Cancer Institute, Xuzhou Medical University, Xuzhou, China; b Center of Clinical Oncology, The Affiliated Hospital of Xuzhou Medical University, Xuzhou, China; c Jiangsu Center for the Collaboration and Innovation of Cancer Biotherapy, Cancer Institute, Xuzhou Medical University, Xuzhou, China; d Center of Animal Laboratory, Xuzhou Medical University, Xuzhou, China

**Keywords:** Albumin-bound paclitaxel (abPTX), NP-abPTX, neutrophil-mediated drug delivery, radiotherapy, myelosuppression

## Abstract

Albumin-bound paclitaxel (abPTX) has been widely used in cancer treatment. However, dose-related side effects, such as myelosuppression, restrict its clinical application. Cell-based targeting drug delivery is a promising way to mitigate systematic side-effects and improve antitumoral efficacy. In this study, we demonstrated that reassembled abPTX could be engulfed by neutrophils *in vivo* and delivered to tumor site, thus improving therapeutic efficacy and mitigating myelosuppression. First, *in vitro* analysis confirmed that reassembling of abPTX formed uniform and stable serum albumin nanoparticles (NP-abPTX) with size of 107.5 ± 2.29 nm and reserved the ability to kill tumor cells. Second, we found that NP-abPTX could be engulfed by activated neutrophil *in vitro* and *in vivo* but do not affect neutrophils’ function, such as chemotaxis and activation. In a murine tumor model, we further proved that local radiotherapy (RT) induced inflammation activated peripheral neutrophils to capture venous infused NP-abPTX and carry them into tumor tissue. As compared to abPTX, infusion of NP-abPTX dramatically enhanced inhibition of tumor growth treated by local RT and mitigated hematotoxicity. Therefore, our study demonstrated a novel strategy to mitigate side-effects and to improve tumor killing efficacy of abPTX through neutrophil-mediated targeting drug delivery.

## Introduction

Paclitaxel (PTX), a member of taxane family, has been widely used for cancer treatment, such as ovarian, breast and non-small cell lung cancers, and malignant brain tumors (Rowinsky & Donehower, 1995; Snyder et al., 2001), low efficacy and serious side effects restrict its application in clinical cancer treatment. To improve therapeutic efficacy and reduce side effects, albumin-bound PTX (abPTX) was created at the right moment, which possesses distinct advantages compared with PTX, including higher dosage and hypoallergenic. However, dose-related side effects of abPTX, especially myelosuppression, are still the insurmountable obstacle for abPTX application partly due to the high-dosage PTX in the circulatory system (Tan et al., 2019). Therefore, new strategies are urgently needed to develop for overcoming the side effects of PTX and improving its therapeutic effect.

Cells-based drug delivery is a promising way to mitigate systematic side-effects and improve therapeutic efficacy of drugs (Su et al., 2015). Neutrophils are abundant and short-lived cells which can be recruited to the inflammatory or infected sites to perform their biological function (Smith, 1994; Brinkmann et al., 2004). Given these characteristics of neutrophils, such as phagocytosis, immediate migration, short life span, and release of contents, neutrophils may be served as an ideal ‘Trojan horse’ to delivery drug into inflammation tissues (Xue et al., 2017). Thus, neutrophil-mediated targeting drug delivery may be attractive in future cancer treatment.

The abPTX is a solvent-free and human albumin-bound nanoparticle with mean size of approximately 130 nm which binds to the albumin receptor of gp60 on endothelial cells to penetrate into surrounding tissues, resulting in more effective utilization (Joerger, 2016), but, this type of nanoparticle still lacks tumor tissue-specific distribution. Studies showed that denatured albumin nanoparticles generated by ethanol and glutaraldehyde can be internalized by activated neutrophils, partly depended on Fcγ receptor III expression (Wang et al., 2014). Hence, reassembling of abPTX into denatured albumin nanoparticles (NP-abPTX) might promote neutrophil-mediated targeting drug delivery to alleviate systemic side-effects and improve the bioavailability and therapeutic efficacy of abPTX through reducing PTX concentration in circulatory system.

Radiotherapy (RT) is a conventional approach for cancer treatment (Yaromina et al., 2012) and significantly improves the survival rate of lung cancer patients (Timmerman et al., 2010). Reports have been proved that RT-induced sterile inflammation induces a rapid activation and tumor infiltration of neutrophils (Takeshima et al., 2016). Therefore, RT is an ideal partner for the neutrophil-mediated drug delivery. Combining these studies, we proposed that local RT-induced sterile inflammation could promote neutrophil activation and uptake of infused NP-abPTX to reduce systemic concentration of PTX and deliver them into irradiated tumor site to enhance the tumor-specific distribution of PTX.

In this study, we reassembled the abPTX to form uniform and stable serum albumin nanoparticles (NP-abPTX) with size of 107.5 ± 2.29 nm. Neutrophil-based NP-abPTX delivery combined with RT remarkably mitigates the myelosuppression complications of abPTX and significantly inhibits the LLC tumor development, when compared with the combination therapy of abPTX and RT. This study provides a novel PTX nanoparticle drug for cancer treatment and the combination therapy of NP-abPTX and RT may be an attractive strategy for tumor treatment.

## Materials and methods

### Preparation of NP-abPTX loaded with or without FITC dye

abPTX (Jiangsu Hengrui Pharmaceuticals Co., Ltd., Lianyungang, China‎) was first dissolved at the concentration of 20 mg/mL in deionized water, and 1 mL abPTX solution was subsequently incubated with the continuously adding 3.5 mL anhydrous ethanol under stirring (1000 rpm) at room temperature for 10 min. For the FITC-labeled nanoparticles, 40 μL FITC dye (5 mg/mL) was added to 1 mL abPTX solution, and the complex was incubated with anhydrous ethanol as the above method. For stabilizing the nanoparticles of abPTX with or without FITC dye, 20 μL 8% glutaraldehyde was added into the solution, which then was allowed to incubation for 4 h at room temperature in the dark. The solution was centrifuged at 12,000 rpm for 15 min at 4 °C, and the suspension was removed. After drying NP-abPTX, the nanoparticles were re-suspended in phosphate-buffered saline (PBS) (G4202, ServiceBio, Wuhan, China) for the next study. Malvern (ZETA SIZER Nano Series, Worcestershire, UK) and transmission electron microscopy (Tecnai G2 Spirit Twin, Thermo Fisher, former FEI, Waltham, MA) were used to detect the size, potential, morphology characteristics of NP-abPTX. The ratio of PTX to human serum albumin is 1/9 in the abPTX according to the drug instructions. For PTX encapsulation, the amount of PTX in total solution was defined as *M*. The total amount of PTX (tP) in the supernatants was detected by high-performance liquid chromatography (HPLC). The PTXs encapsulation efficiency of NP-abPTX=(*M* – tP)**/***M* × 100%.

### Cell culture

LLC cells were obtained from the Procell Life Science & Technology Co., Ltd. (CL-0140, Wuhan, China) and cultured in DMEM medium (KGM12800N-500, KeyGEN, Nanjing, China) with 10% fetal bovine serum (SR100180.03, Sunrise, Claymont, DE) and 1% penicillin–streptomycin (C0222, Beyotime, Shanghai, China) in a 37 °C incubator with 5% CO_2_ atmosphere.

### Cell viability assay

The CCK-8 assay was used to measure the viability of LLC cell treated with abPTX or NP-abPTX. Briefly, LLC cells in the exponential growth phase (3000 cells/well) were cultured in 96-well plates (Corning, Corning, NY) for 24 h, and were then treated with abPTX or NP-abPTX for 48 and 72 h at 37 °C, respectively. Nontreated group and abPTX-treated group were considered as the control group and positive group, respectively. Each group was repeated four times. For detecting the optical density (OD), 10 μL CCK-8 solution (C0038, Beyotime, Shanghai, China) was subsequently added to each group and was incubated with LLC cells for 2 h at 37 °C. The OD value was measured by Cytation 3 Cell Imaging Multi-Mode Reader (BioTek, Winooski, VT) at a wavelength of 450 nm. Relative cell viability was calculated using the following formula: relative cell viability=(mean OD450 of experimental groups/mean OD450 of control groups × 100%).

### Hematological analysis and liver and kidney function detection

Wild type C57BL/6J male mice (6–8 weeks) were purchased from Xuzhou Medical University Animal Center and maintained under standard housing conditions. All animal procedures and protocols are approved by the Experimental Animal Ethics Committee of Xuzhou Medical University, and implemented in accordance with the Xuzhou Medical University Laboratory Animal Care and Use Guide.

The mice of each group (*n* = 4) were irradiated with signal-dose (5 Gy) at the right thigh. After 24 h, the mice were continuously injected via tail vein with abPTX (equivalent 2.7 mg/kg PTX) or NP-abPTX (equivalent 2.7 mg/kg PTX) for five days. Then, the whole blood samples from mice were collected for hematological analysis using Mindray hematologic analyzer (BC-5300, Mindray Bio-Medical Electronics, Shenzhen, China). For liver and kidney function analysis, the blood urea nitrogen (BUN), creatinine (Cre), aspartate aminotransferase (AST), and alanine aminotransferase (ALT) were measured by DRI-CHEM 7000 (FUJI, Tokyo, Japan).

### Phagocytosis assay for NP-abPTX by neutrophils *in vitro*

Neutrophils from C57BL/6J male mouse (6–8 weeks) bone marrow were purified by gradient centrifugation according to the protocol as we previously reported (Wang et al., 2016). The neutrophil purity was detected using PE-mouse-Ly6G (127607, clone 1A8, Biolegend, San Diego, CA) and APC-mouse-CD11b antibody (163702, clone QA19A45, Biolegend, San Diego, CA) by flow cytometry (FCM) analysis (BD FACSCanto II, San Jose, CA) (purity >90%). Neutrophils were cultured in calcium and magnesium-free Hank’s balanced salt solution (HBSS) for the next study.

Neutrophils were cultured in HBSS in six-well plates (Nest, Wuxi, China) at a density of 1 × 10^6^ cells per well. The FITC-labeled NP-abPTX (FITC-NP-abPTX) (50 μg/well) was added into the plates and co-cultured for 0, 1, 2, and 4 h, with or without 50 nM phorbol myristate acetate (PMA) (1652981, Biogems, Westlake Village, CA). Then, neutrophils were collected at the indicated time points, and washed with cold PBS twice. Subsequently, cell samples were incubated with PE-mouse-Ly6G antibody for 30 min at 4 °C in the dark, followed by FCM analysis immediately. The FITC^+^/Ly6G^+^ cells were the neutrophils which phagocytosed FITC-NP-abPTX.

For confocal microscopy assay, neutrophils were labeled with DiI dye (C1036, Beyotime, Shanghai, China) for 5 min, and then were seeded on the glass slide pre-coated with poly-l-lysine (ST509, Beyotime, Shanghai, China) in six-well plates (1 × 10^6^ cells/well) for 4 h. Subsequently, neutrophils were cocultured with FITC-NP-abPTX (50 μg/well) for 1 h. The cells were washed with PBS twice, and then fixed by using 4% paraformaldehyde (VIH100, Servicebio, Wuhan, China) for 20 min in the dark, followed by DAPI staining for nucleus. The location of FITC-NP-abPTX in neutrophil was analyzed by a Zeiss LSM880 confocal laser scanning microscope (Oberkochen, Germany).

### Phagocytosis assay for NP-abPTX by neutrophils *in vivo*

The tumor-free mice were randomly assigned to four groups (*n* = 3) (control, RT, NP-abPTX, and RT + NP-abPTX). The mice of RT and RT + NP-abPTX group were irradiated with signal-dose (5 Gy) at the right thigh. After 24 h, the mice of NP-abPTX and RT + NP-abPTX group were treated with FITC-NP-abPTX (equivalent 2.7 mg/kg PTX) by tail vein injection. Then, the peripheral blood samples of mice were obtained at 1, 2, and 4 h, respectively. After removing red blood cells (RBCs) by RBC lysis buffer (64010-00, Biogems, Westlake Village, CA), the cell samples were washed with cold PBS twice and incubated with PE-mouse-Ly6G for 30 min at 4 °C in the dark. The FITC^+^/Ly6G^+^ cells in peripheral blood were analyzed by FCM analysis to evaluate the NP-abPTX phagocytosis by neutrophil.

For tumor-bearing mice model, LLC cells (3 × 10^5^) in 100 μL PBS were subcutaneously injected on the right thigh of each mouse. When the mean volume of tumors (tumor volume = length × width^2^/2) reached 100 mm^3^, mice were randomized into four groups (*n* = 3) (control, RT, NP-abPTX, and RT + NP-abPTX). The treatment process was the same as described above for tumor-free model and the FITC^+^ neutrophils in peripheral blood were analyzed by FCM analysis.

### Migration assay

Neutrophils were pre-cultured with or without 50 μg NP-abPTX for 1 h. Then, the neutrophils were seeded in the upper transwell chambers with 3 μm pore size (353096, Corning, Glendale, AZ) at a density of 1 × 10^6^ cells per well in 100 μL serum-free medium with 100 nM N-formylmethionyl-leucyl-phenylalanine (fMLP) supplemented medium in the lower chambers as chemoattractant. After 30 min incubation, the cells in the upper chambers were removed. The cells that had passed through the membrane were photographed and counted.

### Reactive oxygen species (ROS) detection assay

Neutrophils, isolated as above, were pre-cultured with or without 50 μg NP-abPTX for 30 min, and then were incubated with 10 mg/mL H_2_DCFDA (D399, Thermo Fisher Scientific, Waltham, MA) for 30 min. For positive control, 100 nM PMA was added to the corresponding groups for 10 min. The ROS production was measured by FCM analysis and Cytation 3 Cell Imaging Multi-Mode Reader (BioTek, Winooski, VT) immediately.

### Real-time PCR

Neutrophils, isolated as above, were pre-cultured with or without 50 μg NP-abPTX for 30 min. For positive control, 50 nM PMA was added to the corresponding groups for 30 min. Total RNA was extracted from the cells using TRIzol Reagent (15596-026, Invitrogen, Waltham, MA) following the manufacturer’s instruction. First-strand cDNA synthesis was conducted using the HiScript^®^ Q RT SuperMix for qPCR (+gDNA wiper) kit (R123-01, Vazyme, Nanjing, China) according to the manufacturer's protocol. For analyzing the mRNA expression levels of *Il-1β*, *Il-6*, *Nos2*, and *Gapdh*, RT-qPCR analysis was performed on the LightCycler^®^ 480 Real-Time PCR System (Roche, Mannheim, Germany) in a 20 µL reaction mixture including 10 µL AceQ^®^ qPCR SYBR Green Master Mix (Q111-02, Vazyme, Nanjing, China), 0.8 µL primer mixture (forward and reverse, 10 µM), 1 µL cDNA, and 8.2 µL diethyl pyrocarbonate-treated water. According to the manufacturer's protocol, the thermocycling profile consisted of 95 °C for 5 min, followed by 40 cycles at 95 °C for 10 s and 60 °C for 30 s. *Gapdh* was used as an internal control. The experiment was performed three times and data were analyzed using the 2^–ΔΔCt^ method. The primer sequences were as follows: *Il-1β* forward, 5′-CGACAAAATACCTGTGGCCT-3′ and reverse, 5′-TTCTTTGGGTATTGCTTGGG-3′; *Il-6* forward, 5′-TCCAGTTGCCTTCTTGGGAC-3′ and reverse, 5′-GTGTAATTAAGCCTCCGACTTG-3′; *Nos2* forward, 5′-CTGCAGCACTTGGATCAG-3′ and reverse, 5′-CGTACCAGGCCCAATGAG-3′; and *Gapdh* forward, 5′-TGCGACTTCAACAGCAACTC-3′ and reverse, 5′-CTTGCTCAGTGTCCTTGCTG-3′.

### Efficacy and safety evaluation of NP-abPTX combined with local radiotherapy

LLC cells (3 × 10^5^) in 100 μL PBS were subcutaneously injected on the right thigh of each mouse. When the mean volume of tumors reached 100 mm^3^, mice were randomized into five groups (*n* = 6) (PBS, abPTX, RT, RT + abPTX, and RT + NP-abPTX). Mice of RT, RT + abPTX, and RT + NP-abPTX group were treated with tumor local RT with signal-dose (5 Gy) twice (three days apart), and the mice of RT + abPTX and RT + NP-abPTX group were administrated with abPTX (equivalent 2.7 mg/kg PTX) and NP-abPTX (equivalent 2.7 mg/kg PTX) by i.v., respectively, for three consecutive days after every RT treatment. At the corresponding time points, mice of PBS and abPTX group were treated with PBS and abPTX (equivalent 2.7 mg/kg PTX), respectively. In the process, the mice weight and tumor volume were monitored. The mice were sacrificed at 27th day, whose tumor tissues were weighed and harvested for later experiments.

For evaluating the sub-acute toxicity of NP-abPTX, normal organs of mice were harvested and fixed with 4% paraformaldehyde, then were stained with hematoxylin–eosin (H&E) and visualized by inverted fluorescence microscope (IX83, Olympus, Tokyo, Japan). For NP-abPTX positive neutrophil detection in tumors, the fresh tumor tissues were ground into single cell suspensions. Then, the single cell suspension was filtrated with 40 μm nylon filter membrane (352340, Corning, Glendale, AZ). The RBCs were removed by RBC lysis buffer. The single cell suspension was washed with cold PBS twice and collected. The cell pellet was resuspended with cold PBS and stained with PE-mouse-Ly6G for 45 min at 4 °C in the dark. The cell samples were analyzed by FCM to determine the proportion of NP-abPTX positive cell in neutrophils and tumor cells.

### Evaluation of neutrophil’s role in the combination treatment of NP-abPTX and radiotherapy

The LLC-bearing mice model was generated as mentioned above. When the mean volume of tumors reached 100 mm^3^, mice were randomized into two groups (*n* = 4) (RT + anti-Ly6G and RT + anti-Ly6G + NP-abPTX). The mice were given intraperitoneal injection of anti-mouse Ly6G antibody (Clone 1A8, Biolegend, San Diego, CA) (125 μg/20 g), followed by tumor local RT with signal-dose (5 Gy), and then the mice of NP-abPTX-treated group were administrated with NP-abPTX (equivalent 2.7 mg/kg PTX) by tail vein injection for three consecutive days. This treatment procedure was repeated twice. In the process, the mice weight and tumor volume were recorded. The mice were sacrificed at 27th day, whose tumor tissues were weighed.

### Statistical analysis

All data are presented as the mean ± standard deviation (SD) unless otherwise stated. Statistical analyses were performed using Prism v.7.0 (GraphPad Software, La Jolla, CA). The significance of the difference between two independent samples was determined using unpaired Student’s *t*-test. One-way ANOVA with Tukey’s test was used to compare multiple groups. A value of *p*< .05 was considered statistically significant for all experiments.

## Results

### NP-abPTX maintains antitumoral effect *in vitro*, similar to abPTX

**Figure 1.**
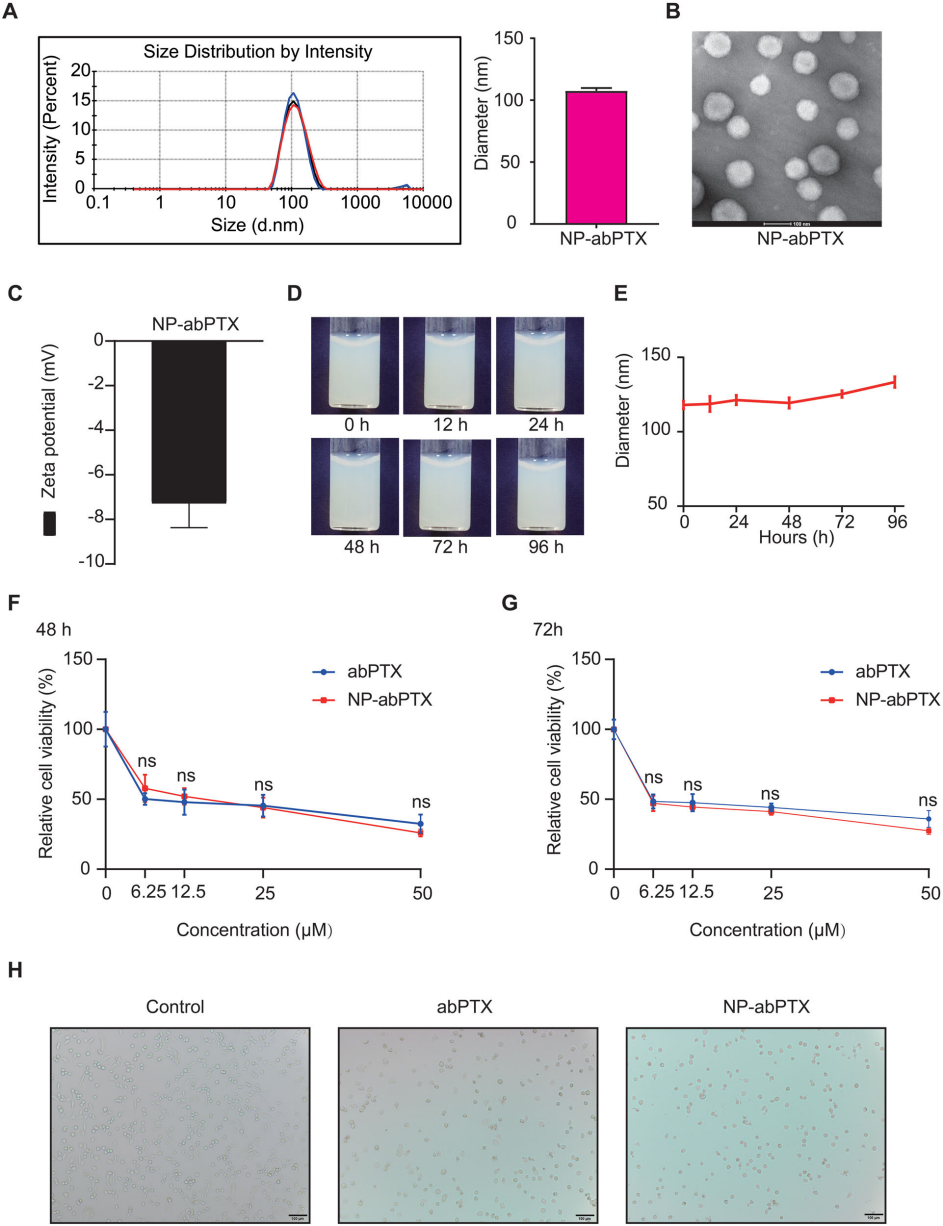
NP-abPTX maintains anti-proliferation effect *in vitro*, similar to abPTX. (A) Size intensity curves and the mean size of NP-abPTX. (B) The shapes of NP-abPTX were acquired by transmission electron. Scale bars, 100 nm. (C) Surface zeta potential. (D) The NP-abPTX suspensions were photographed at indicated time. (E) The NP-abPTX size was measured by Malvern at indicated time. (F, G) CCK-8 assay was used to determine the anti-proliferation effects of abPTX and NP-abPTX on LLC cells. Each group was repeated four times; ns: not significant. (H) The morphology changes of LLC cells treated by 6.25 μM abPTX or NP-abPTX for 48 h. Representative pictures are presented.

The NP-abPTX was obtained by modifying abPTX in the manner described above (Wang et al., 2014). We measured the basic characteristics of NP-abPTX, including size, potential, and shape. As shown in Figure 1(A), the mean diameter of NP-abPTX was 107.5 ± 2.29 nm. The shape of NP-abPTX was spherical (Figure 1(B)). Figure 1(C) shows that the mean zeta potential of NP-abPTX was −7.28 ± 1.1 mV. The NP-abPTX maintained good stability in PBS for at least four days (Figure 1(D,E)). These data showed that abPTX was successfully modified to NP-abPTX with main size of 107.5 nm and good stability. The results of HPLC indicated that the loading ratio of PTX was about 40%, compared to the amount of PTX in abPTX. According to this ratio, the dosage of NP-abPTX in subsequent experiments was determined by the amount of PTX.

To determine whether reassembled NP-abPTX restrained the antitumoral ability, we used a CCK-8 assay to evaluate the antiproliferative effect of NP-abPTX on LLC cells. As shown in Figure 1(F,G), NP-abPTX could inhibit the viability of LLC cells, similar to abPTX. Morphological detection showed that treatment with NP-abPTX or abPTX obviously caused a spread-to-round morphology change at the concentration of 6.25 μM for 48 h, when compared with control group (Figure 1(H)). These results indicated that NP-abPTX could inhibit the proliferation for LLC cells *in vitro*, similar to abPTX.

### NP-abPTX alleviates myelosuppression

**Figure 2.**
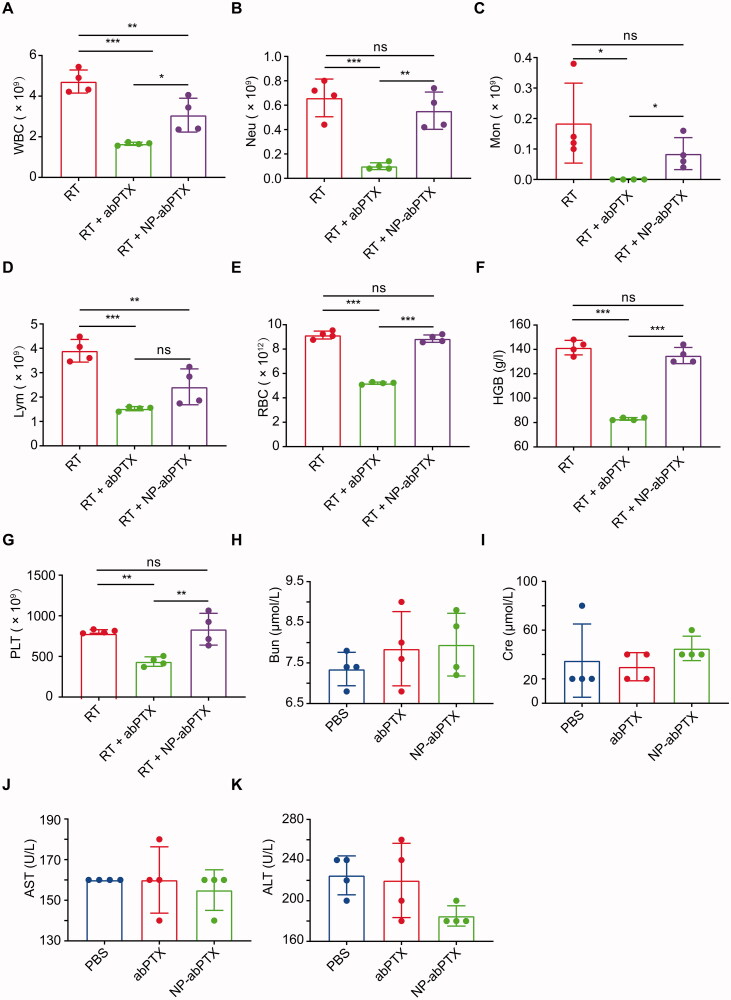
The NP-abPTX administration induces lower myelosuppression side effects than abPTX and does not lead to the serious damage of liver and kidney function. Based on the local RT, for combination therapy, mice were treated with abPTX or NP-abPTX (equivalent 2.7 mg/kg PTX) for five days, and subsequently the peripheral blood samples of mice were taken for testing. (A) White blood cell. (B) Neutrophil. (C) Monocyte. (D) Lymphocyte. (E) Red blood cell. (F) Hemoglobin. (G) Platelet. Mice were treated with PBS, abPTX, and NP-abPTX (equivalent 2.7 mg/kg PTX) for five days, respectively, and subsequently the serum samples of mice were taken for testing. (H) Blood urea nitrogen. (I) Creatinine. (J) Aspartate aminotransferase. (K) Alanine aminotransferase. Data are presented as the mean ± SD, *n* = 4 (*p<.05, **p< .01, ***p< .001; ns: not significant).

The previous study showed that albumin-based nanoparticles generated by ethanol and glutaraldehyde can be loaded by activated neutrophils (Wang et al., 2014). We speculated that NP-abPTX, created by above method, can also be internalized by activated neutrophils, which may reduce PTX concentration in circulatory system, thereby attenuating the myelosuppression side-effects. To confirm this speculation, we performed the hematological analysis after infusion of PBS, NP-abPTX, or abPTX in a RT-induced sterile inflammation model. As compared to control group, infusion of abPTX significantly decreased white blood cell (WBC) count, including neutrophils, monocytes, and lymphocytes (Figure 2(A–D)). Moreover, RBC, hemoglobin (HGB) and platelet (PLT) were also obviously declined after abPTX administration (Figure 2(E–G)). Not surprisingly, abPTX treatment caused severe hematotoxicity as reported before. In turn, administration of reassembled NP-abPTX dramatically mitigated the hematotoxicity compared with abPTX (Figure 2(A–G)). Taken together, these data indicated that reassembling of abPTX into NP-abPTX could alleviate the myelosuppression complications.

Dysfunctions of liver and kidney are always another common adverse reaction for chemotherapy (Superfin et al., 2007; Ramadori & Cameron, 2010). Thus, we further evaluated the effect of NP-abPTX on the functions of liver and kidney. We found no statistical significance between each group for BUN, Cre, AST, and ALT (Figure 2(H–K)), suggesting that NP-abPTX does not cause damage to liver and kidney. Collectively, these data indicated that NP-abPTX could significantly attenuate chemotherapy-induced myelosuppression without detectable abnormal function of liver and kidney.

### NP-abPTX can be internalized by neutrophils *in vitro*

**Figure 3.**
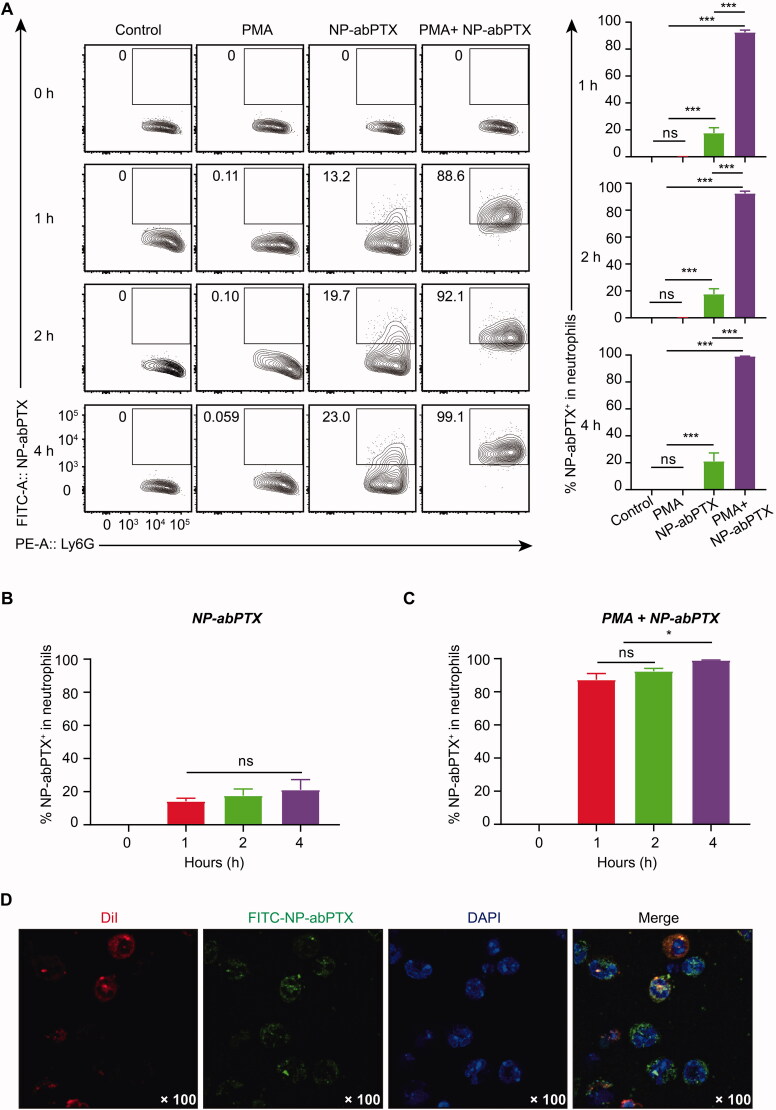
NP-abPTX can be internalized by activated neutrophil *in vitro*. Mouse neutrophils were incubated with FITC-NP-abPTX for 0, 1, 2, and 4 h, respectively, with or without 50 nM PMA. (A) Flow cytometry was used to determine the FITC^+^/Ly6G^+^ neutrophils. Representative results are presented (*n* = 3). Corresponding statistic results of (A) are presented at right side of the flow graph (***p< .001; ns: not significant). (B) The neutrophils were cocultured with FITC-NP-abPTX. The FITC^+^ neutrophils were measured by flow cytometry analysis at the indicated time. (C) The neutrophils were cocultured with 50 μg FITC-NP-abPTX and 50 nM PMA. The FITC^+^ neutrophils were measured by FCM analysis at indicated time (*p< .05; ns: not significant). (D) Internalization of FITC-NP-abPTX in neutrophils is detected by confocal microscopy at ×100 magnification. Representative pictures are presented.

Neutrophils were cultured with FITC-labeled NP-abPTX for 0, 1, 2, and 4 h at 37 °C to confirm whether NP-abPTX can be engulfed by neutrophils. The FITC^+^/Ly6G^+^ subset was considered as NP-abPTX containing neutrophils. As shown in Figure 3(A), the ratio of FITC^+^/Ly6G^+^ subset was significantly increased after co-cultivation of NP-abPTX and neutrophils for 1, 2, and 4 h, when compared with the control group at the corresponding time point (*p*< .001). Activation of neutrophils by PMA further enhanced uptake of NP-abPTX (Figure 3(A)). In addition, we evaluated the influence of co-cultivation time on the NP-abPTX phagocytosis by neutrophils. As shown in Figure 3(B), the ratio of FITC^+^/Ly6G^+^ subset was not different in un-stimulated neutrophils between different time points. However, the FITC^+^/Ly6G^+^ subset ratio at 4 h in PMA-stimulated neutrophils was significantly higher than that at 1 and 2 h (*p*< .05) (Figure 3(C)), indicating that continuous inflammation stimulation promotes neutrophils to phagocytose NP-abPTX.

To further confirm that NP-abPTX could be internalized by neutrophils, confocal microscopy imaging was performed to visualize internalized NP-abPTX in neutrophils. As shown in Figure 3(D), FITC-NP-abPTX was located in the neutrophil cytoplasm. Above all, these data showed that NP-abPTX can be loaded by neutrophils *in vitro*, especially activated neutrophils.

### Infused NP-abPTX can be captured by neutrophils *in vivo*

**Figure 4.**
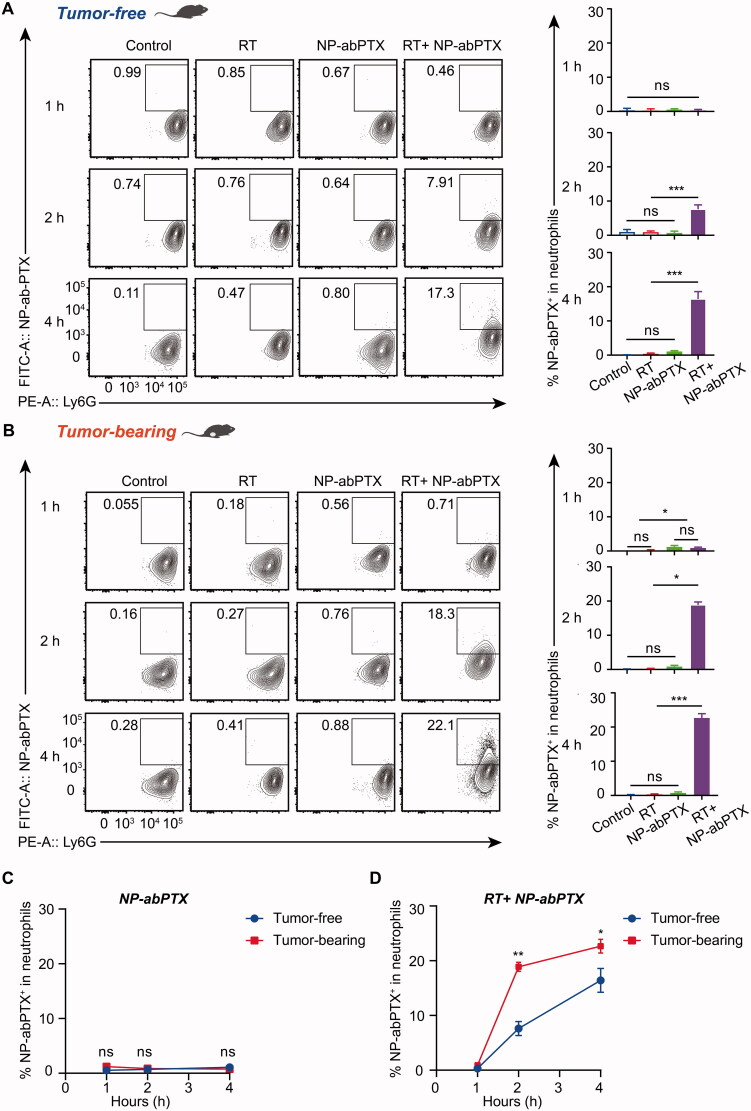
NP-abPTX can be internalized by RT-activated neutrophil *in vivo*. Tumor-free mice were irradiated with signal-dose (5 Gy) at the right thigh, followed by NP-abPTX administration after 24 h. Flow cytometry was used to measure the FITC^+^/Ly6G^+^ neutrophils at the indicated time. (A) RT-activated neutrophils can phagocytose FITC-NP-abPTX at 2 and 4 h after drug administration. LLC-bearing mice were treated by the same procedure as above. (B) the FITC^+^/Ly6G^+^ neutrophils were detected by flow cytometry at 1, 2, and 4 h after drug administration. Representative results are presented (*n* = 3). Corresponding statistic results of (A) and (B) are presented at right side of the flow graph (*p< .05, ***p< .001; ns: not significant). (C) Comparison of FITC^+^ neutrophil between tumor-free and tumor-bearing mice without RT. (D) Comparison of FITC^+^ neutrophil between tumor-free and tumor-bearing mice with RT (*p< .05; ns: not significant).

To further confirm whether NP-abPTX can be captured by neutrophils *in vivo*, we evaluated the percentage of NP-abPTX^+^/Ly6G^+^ neutrophils in peripheral blood in tumor-free and tumor-bearing mice with or without local RT. The right thigh of tumor-free or tumor-bearing mice was irradiated with a single-dose of 5 Gy, followed by tail vein injection of FITC-labeled NP-abPTX. At the timepoints of 1, 2, and 4 h after injection, peripheral blood samples were collected and used for FCM assay. FITC and Ly6G double-positive cells were considered as neutrophils carrying NP-abPTX. As shown in Figure 4(A), very few neutrophils were detected positive for FITC without RT induction, while treatment with RT significantly promoted capture of NP-abPTX by neutrophils. Moreover, in LLC tumor-bearing mice, as shown in Figure 4(B), combination treatment of RT and NP-abPTX significantly increased the percentage of FITC^+^/Ly6G^+^ subset compared with NP-abPTX treatment at 2 h (*p*< .05) and 4 h (*p*< .001), respectively.

To confirm whether tumor-bearing would affect capture of NP-abPTX by neutrophils, we compared the percentage of FITC-positive neutrophils in tumor-free and tumor-bearing mice and found no statistical significance in NP-abPTX group (Figure 4(C)). Interestingly, RT treatment induced sterile inflammation significantly promoted neutrophil activation and thus engulfing of NP-abPTX. As shown in Figure 4(D), more neutrophils carrying NP-abPTX were detected in tumor-bearing mice than in tumor-free mice after RT treatment. These data indicated that continuous inflammatory stimulation induced by RT could promote uptake of NP-abPTX by neutrophils.

### Neutrophil function is not affected by engulfed NP-abPTX

**Figure 5.**
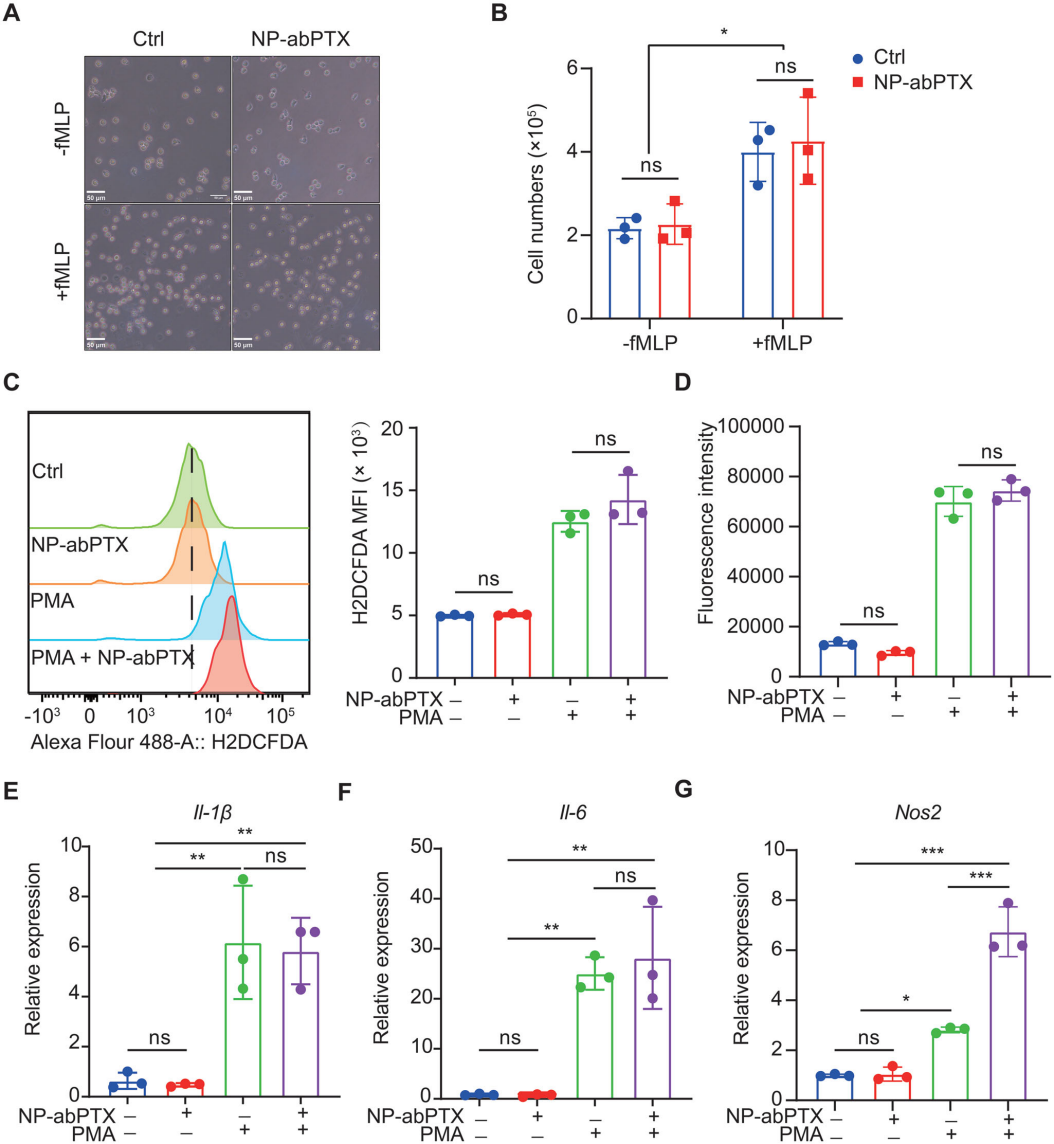
NP-abPTX does not affect neutrophil’s function. (A) Transwell migration assay for neutrophils pre-cultured with or without 50 μg NP-abPTX. Cells that had migrated to the bottom chamber were photographed. The experiment was repeated three times and representative pictures are presented. Scale bars, 50 μm. (B) Total numbers of neutrophils in the lower chambers. (C) ROS levels of neutrophils measured by flow cytometry. (D) ROS levels of neutrophils measured by Cytation 3 Cell Reader. (E–G) Reverse transcription-quantitative polymerase chain reaction analyses of *Ii-1β*, *Ii-6*, and *Nos2* mRNA expression. The experiment was repeated three times, and *Gapdh* was regarded as a loading control. Data are presented as the mean ± SD, *n* = 3 (*p< .05, **p< .01, ***p< .001; ns: not significant).

To determine whether neutrophils’ function would be affected by engulfed NP-abPTX, cell migration, respiratory burst, and inflammatory gene expression of neutrophils were detected. Since cell motility is essential for neutrophil-mediated targeting drug delivery (Wang et al., 2014; Xue et al., 2017), transwell migration assay was performed to detect neutrophil chemotaxis. Upon stimulation by chemoattractant fMLP, neutrophils quickly transmigrated into the lower chamber. Meanwhile, the transmigration was not affected by NP-abPTX loading (Figure 5(A,B)).

ROS is critical for neutrophils to eliminate invading microbial pathogens (Bokoch & Zhao, 2006). For evaluating the influence of NP-abPTX on neutrophil ROS production, intracellular ROS was measured by H2DCFDA fluorescence using FCM (Figure 5(C)) or Cytation 3 Cell Imaging Multi-Mode Reader (Figure 5(D)), respectively. As shown in Figure 5(C,D), ROS production in neutrophils was not induced by treatment with NP-abPTX only. While PMA stimulation significantly promoted neutrophil ROS generation and loading of NP-abPTX did not have a significant influence on PMA-induced neutrophil ROS production.

In addition, representative inflammatory gene expression of neutrophil was further evaluated. As shown in Figure 5(E,F), no difference in the expression of *Il-1β* and *Il-6* was found between NP-abPTX treated group and untreated group, irrespective of PMA stimulation. For inducible nitric oxide synthetase (*Nos2*) (Figure 5(G)), uptake of NP-abPTX significantly improved the *Nos2* expression in neutrophils compared with PMA group (*p*< .05), but NP-abPTX alone had no effect on the *Nos2* expression compared with untreated group. Taken together, these data demonstrated that NP-abPTX has no obvious affection on neutrophil’s function, such as cell migration, ROS production, and inflammatory gene expression.

### Local radiotherapy induces neutrophil activation and targeting delivery of NP-abPTX thus improving therapeutic efficacy

**Figure 6.**
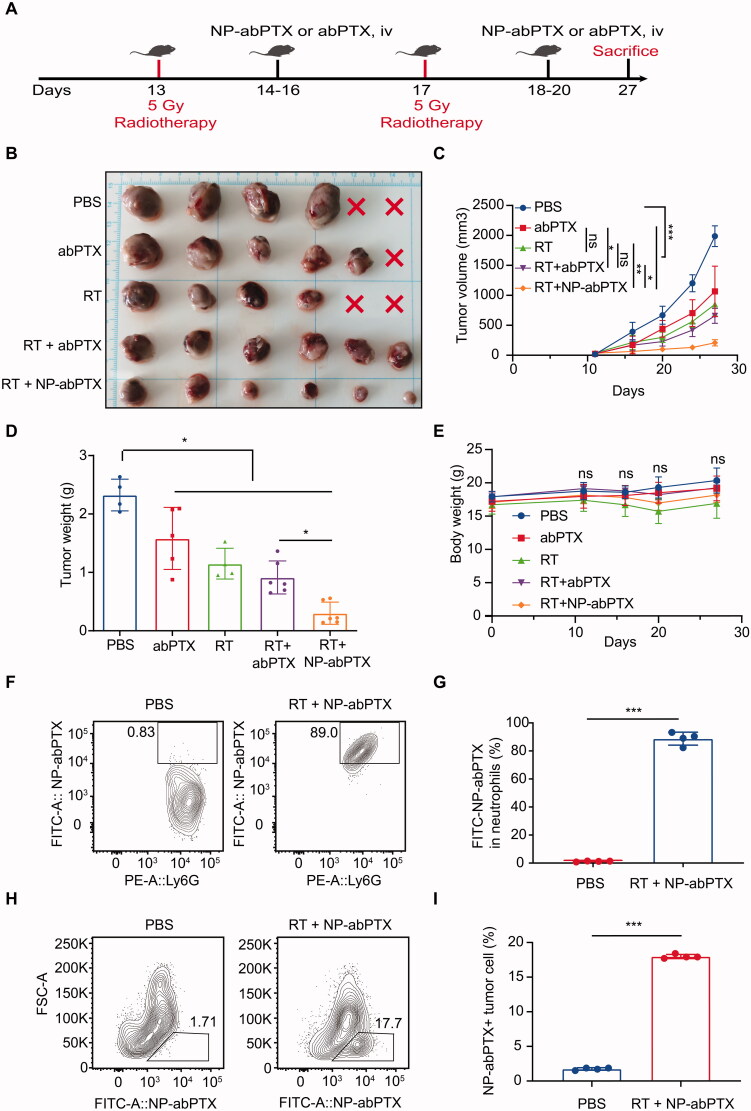
Neutrophil-medicated targeting NP-abPTX delivery suppresses the progression of LLC subcutaneous tumor. (A) Schema of the subcutaneous LLC tumor model treated with RT, followed by NP-abPTX or abPTX administration. (B) Tumor size. The red cross marks represent the mice that died of tumor during the experiment. (C) Tumor volume. (D) Tumor weight. (E) Body weight. (F) Ratio of FITC-NP-abPTX^+^ neutrophil in tumor-infiltrating neutrophils. (G) Statistic results of (F). (H) Ratio of FITC-NP-abPTX^+^ tumor cells. (I) Statistic results of (H). Data are presented as the mean ± SD (*p< .05, ***p< .001; ns: not significant).

Our previous data have proved that NP-abPTX could be engulfed by activated neutrophils. As reported, local RT-induced aseptic inflammation could promote neutrophil infiltration into tumor tissues (Takeshima et al., 2016). Accordingly, we speculated that combining RT with NP-abPTX may have a better anti-tumor effect. Schematic diagram of experimental procedure is shown in Figure 6(A). LLC tumor-bearing mice were treated with RT (5 Gy) on the 13th and 17th day after tumor inoculation, followed by infusion of NP-abPTX or abPTX for three consecutive days. Results showed that tumor growth was significantly prevented with treatment of abPTX, RT, RT + abPTX, or RT + NP-abPTX as compared to PBS group (Figure 6(B,C)). Within the RT combination groups, the average tumor volume in the NP-abPTX group on day 27 was 206.25 ± 53.77 mm^3^, and the mean tumor volume in abPTX was 656.73 ± 124.47 mm^3^, the therapeutic effect of RT + NP-abPTX was significantly superior to RT + abPTX (*p*< .05; Figure 6(C)). As shown in Figure 6(D), the wet weights of tumors were significantly lower in the treated groups when compared with the PBS group, and the tumor weight of RT + NP-abPTX group was significantly lower than that of RT + abPTX group (*p*< .05). Among those treatments, combining RT with NP-abPTX presented best therapeutic efficacy.

To preliminarily evaluate the side-effects in different treatment groups, body weight of mice was measured. We found that there was no statistical significance between each group (Figure 6(E)), suggesting that mice in each group were able to tolerate the treatments. To confirm whether NP-abPTX could be delivered into tumor tissues by neutrophils, FITC-NP-abPTX positive cells in tumor tissues were analyzed by FCM. Figure 6(F–G) indicates that most of the neutrophils in tumor tissues from RT + NP-abPTX treated group were FITC positive, indicating that neutrophils could deliver NP-abPTX into tumor site. Furthermore, we found that NP-abPTX could be absorbed by tumor cells (Figure 6(H,I)), indicating that NP-abPTX could be released by neutrophil to kill tumor cells. Collectively, these data suggested that NP-abPTX holds a better anti-tumor effect than abPTX in the LLC-bearing mice *in vivo*, when combined with RT.

**Figure 7.**
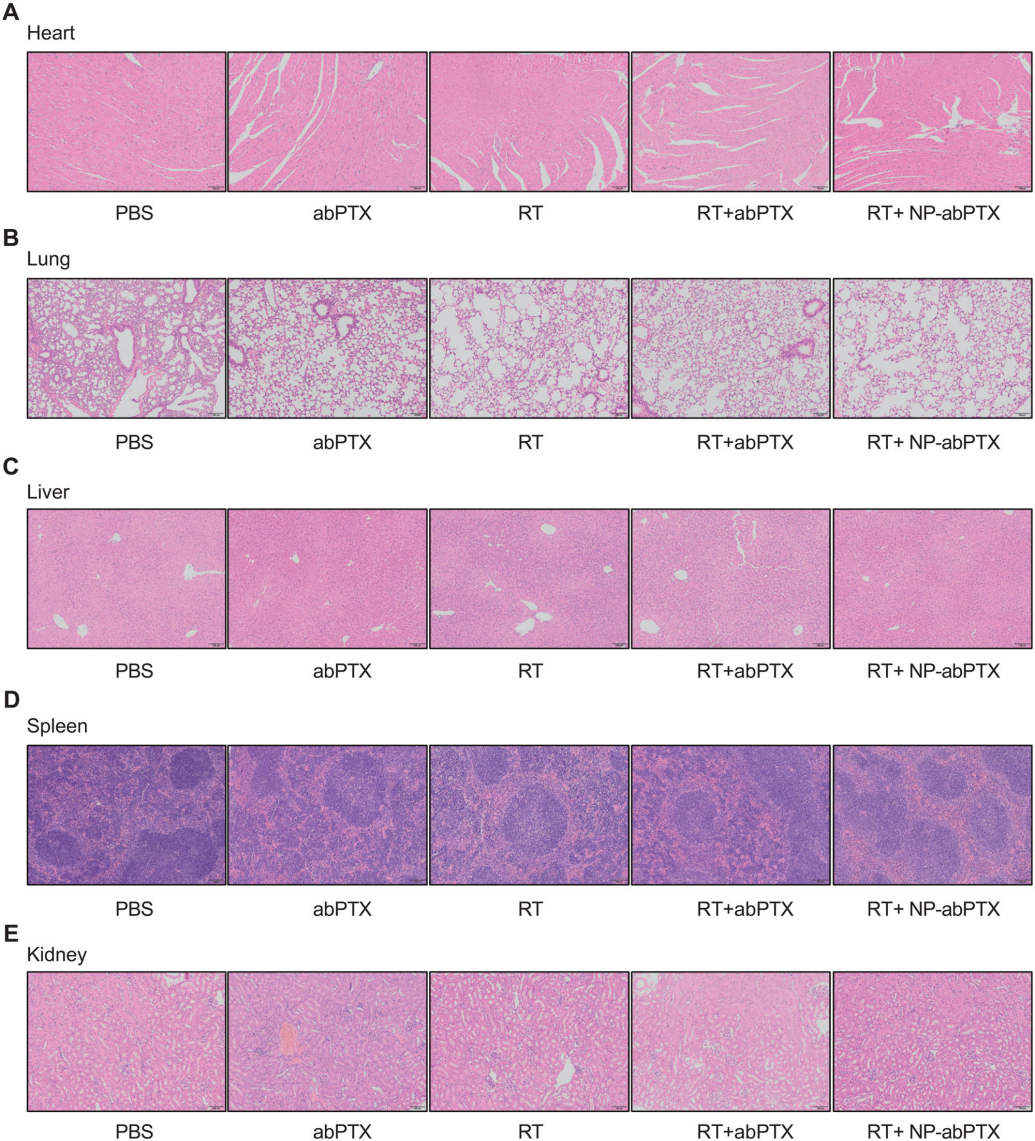
NP-abPTX has no obvious toxic effect to the main organs of mice. (A) H&E staining for heart. (B) H&E staining for lung. (C) H&E staining for liver. (D) H&E staining for spleen. (E) H&E staining for kidney. Scale bars, 100 μm.

In addition, *in vivo* safety evaluation of NP-abPTX was performed to test the sub-acute toxicity by multi-organ pathological assay. Hematoxylin–eosin staining for paraffin sections of heart, lung, liver, spleen, and kidney showed no apparent morphological changes in treated groups, with normal nuclear morphology, complete tissue structure, and no obvious inflammatory infiltration, when compared with the control group (Figure 7). These data suggested that the combination treatment of NP-abPTX and RT significantly suppresses the progression of LLC, and has no obvious sub-acute toxicity.

### Antitumoral activity of NP-abPTX *in vivo* is neutrophil-dependent

**Figure 8.**
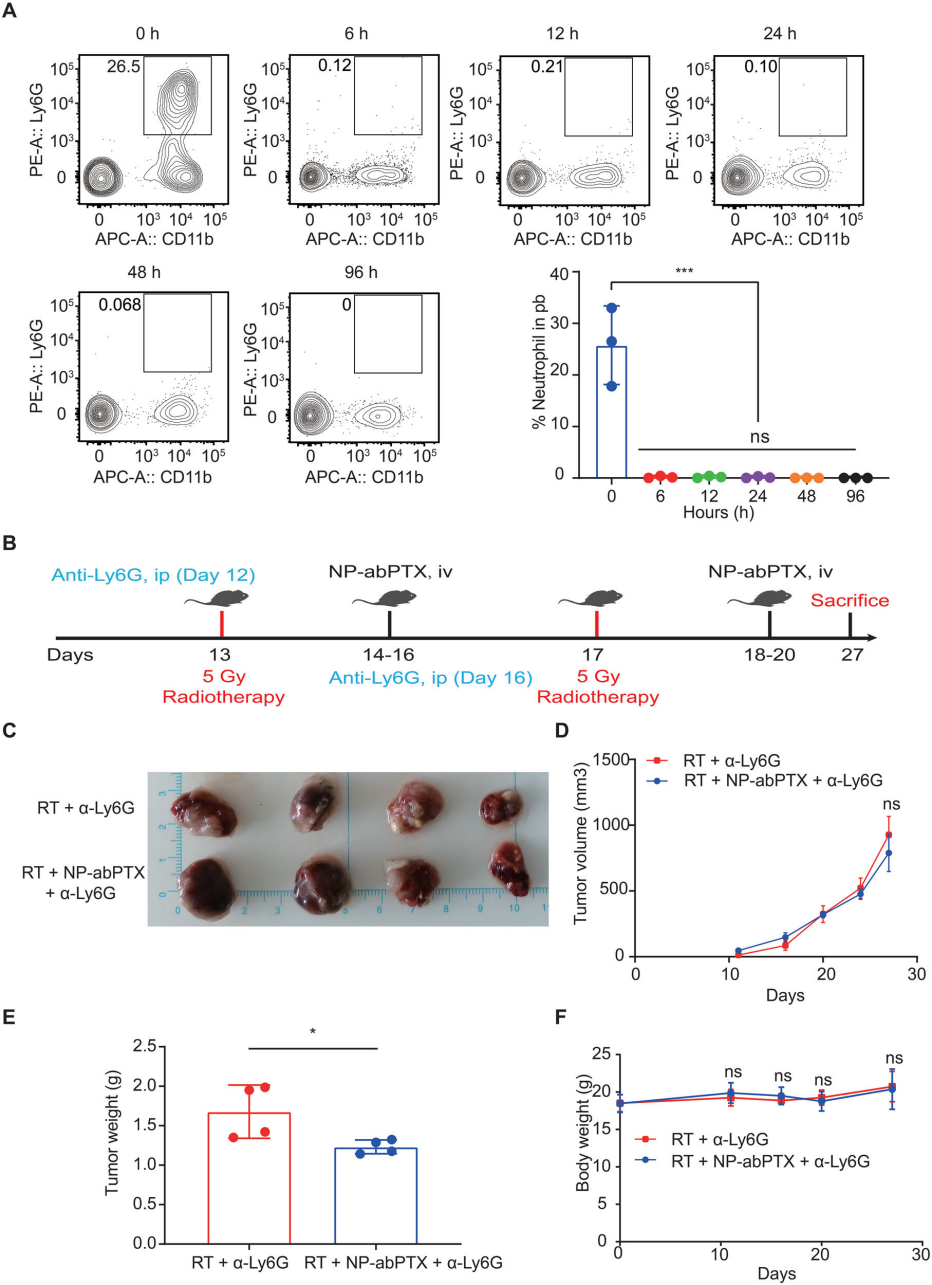
Antitumoral activity of NP-abPTX *in vivo* is neutrophil-dependent. (A) The depletion effect of anti-Ly6G antibody *in vivo*. (B) Schema of the subcutaneous LLC tumor model for evaluating the role of neutrophil in the combination therapy of NP-abPTX and RT. (C) Tumor size. (D) Tumor volume. (E) Tumor weight. (F) Body weight. Data are presented as the mean ± SD, *n* = 4 (*p< .05, ns: not significant).

Our previous data found that NP-abPTX can be loaded and be transported into tumor site via RT-activated neutrophils to exert anti-tumor effect. To further confirm neutrophils are indispensable for targeting delivery of NP-abPTX, neutrophils were depleted with intraperitoneal injection of anti-Ly6G antibody. As shown in Figure 8(A), neutrophils in circulatory system could be eliminated at 6 h after anti-Ly6G antibody administration, and the depletion effect could be maintained for at least four days.

Next, we reevaluated the combination effect between NP-abPTX and RT, under the setting of neutrophil depletion. As shown in Figure 8(B), tumor-bearing mice were received anti-Ly6G antibody administration on the days (12 and 16) before RT (5 Gy), and the NP-abPTX treatment on the days (14–16; 18–20) after RT (5 Gy). Although the wet tumor weight of RT + NP-abPTX + a-Ly6G was lower than RT + a-Ly6G (*p*< .05) (Figure 8(E)), we found no statistical significance on the tumor size and tumor volume between RT + a-Ly6G and RT + NP-abPTX + a-Ly6G (Figure 8(C,D)). Meanwhile, we found no statistical significance on body weights of mice between above two groups (Figure 8(F)). Taken together, these data suggested that the combination effect between RT and NP-abPTX is depended on neutrophils.

## Discussion

Although abPTX possesses better efficiency and lower toxicity in lung cancer treatment than solvent-based PTX, the adverse effects remain one of the major obstacles for abPTX application, such as neutropenia, neuropathy, fatigue, and thrombocytopenia (Socinski et al., 2010). Moreover, a system review and meta-analysis demonstrated that lung cancer patients treated with abPTX experienced more obvious side effects than those patients treated with traditional solvent-based PTX, especially anemia and thrombocytopenia (Tan et al., 2019). Therefore, retaining antitumoral efficacy and mitigating side effects of abPTX will be conducive to extend its clinical application and improve survival quality of cancer patients. In the present study, we demonstrated that reassembled abPTX (NP-abPTX) can be transported into irradiated tumor site through neutrophil-mediated targeting delivery thus alleviating hematotoxicity and improving therapeutic efficacy.

Cell-based drug delivery is a promising way to mitigate systemic side effects and improve therapeutic effect of chemical drugs (Pierige et al., 2008; Gutiérrez Millán et al., 2012; Yu et al., 2020). More importantly, blood cells including erythrocytes, PLTs and leukocytes naturally have the ability to cross blood brain barriers which have been used for drug delivery (Xue et al., 2017; Li et al., 2021). Neutrophils are the most abundant circulating leukocytes activated quickly and recruited to the inflammatory or infection sites the first time. Studies have proved that neutrophils can phagocytose several kinds of nanoparticles as a ‘Trojan horse’ to delivery drugs (Wang et al., 2014; Chu et al., 2017; Villanueva, 2017; Xue et al., 2017; Hao et al., 2020; Naumenko et al., 2020). By reducing systemic toxic side effects, enhancing targeting and improving the penetrability of drugs to tumor cells, neutrophil-mediated targeting drug delivery exhibits great potential for clinical application in the future.

Recent study found that abPTX (commercial name: Abraxane) can be loaded into neutrophils *in vitro* and thus improving therapeutic efficacy for murine gastric cancer (Ju et al., 2019). However, neutrophils are short-lived cells and *in vitro* drug loading might cause serious cell death which is harmful for recipient. Moreover, *in vitro* drug loading in neutrophils may also increase risk of infection. Denatured albumin nanoparticles prepared via desolvation method can be specifically internalized by activated neutrophils *in vivo* and delivered to inflammation or tumor tissues (Wang et al., 2014; Chu et al., 2015). In the present study, abPTX was reassembled to form denatured albumin nanoparticles of NP-abPTX that was proved to be captured by activated neutrophils *in vitro* and *in vivo*. Sterile inflammation induced by local RT could activate and induce neutrophils to migrate into tumor tissue (Takeshima et al., 2016). Accordingly, we speculated that reassembling of abPTX to acquire denatured albumin nanoparticles (NP-abPTX), which could be engulfed by local RT-activated neutrophils, might alleviate systemic side effects and promote tumor targeting drug delivery.

*In vitro* and *in vivo* experiments have confirmed that reassembling of abPTX into NP-abPTX maintains its antitumoral property of PTX. Interestingly, NP-abPTX treatment kills tumor cells while does not affect neutrophil function. In common with other cytotoxic chemotherapy drugs, PTX preferentially attack fast-proliferating cells. Since neutrophils are terminally differentiated cells with a short lifespan. That is probably why neutrophils are not affected by PTX. To further determine the role of neutrophils in the treatment process, we evaluated the combination treatment efficacy of NP-abPTX and RT under the setting of neutrophil depletion by anti-mouse Ly6G antibody. Results showed that there was no significant difference in the tumor volume between the two groups (Figure 8(D)), indicating that neutrophils did play a critical role in delivery of NP-abPTX into tumors where NP-abPTX exerts its antitumoral function.

The combination therapy of NP-abPTX and RT effectively attenuated the progression of LLC tumor *in vivo*; however, several limitations are still existed. First, these characteristics (the mean diameter = 107.5 ± 2.29 nm, the main zeta potential= −7.28 ± 1.1 mV, and neutrophils-mediated endocytosis) may protect the NP-abPTX from being engulfed by reticuloendothelial system (RES) to some extent, but, NP-abPTX may still be partly cleared by RES which may restrict the therapeutic efficacy of NP-abPTX. Second, the efficacy of abPTX alone is weaker than other studies. We speculated that the main reason should be the low dosage of PTX we used in the study. Moreover, the different sources of abPTX may also cause the delicate differences in tumor controlling efficacy.

Collectively, in the present study, we demonstrated that neutrophil mediated NP-abPTX delivery, induced by tumor local RT, significantly suppresses the LLC progression with less side effects. The combination of local RT and NP-abPTX infusion might be an attractive strategy for cancer treatment.
